# EUS-Anchored Multimodal Evaluation of Pancreatic Cystic Lesions: Toward a Conceptual Diagnostic Framework

**DOI:** 10.3390/jcm15103893

**Published:** 2026-05-18

**Authors:** Enshuo Liu, Fei Yang

**Affiliations:** Endoscopy Center, Shengjing Hospital of China Medical University, Shenyang 110004, China; lakeblue18@163.com

**Keywords:** endoscopic ultrasound, pancreatic cystic lesions, CE-EUS, elastography, CEA, KRAS, NGS, artificial intelligence

## Abstract

Pancreatic cystic lesions (PCLs) represent a growing clinical challenge due to their diverse biological behaviors and the substantial overlap in imaging features between benign, premalignant, and malignant entities. Traditional diagnostic approaches relying on cross-sectional imaging or isolated morphologic criteria frequently fail to achieve adequate risk discrimination. Advances in endoscopic ultrasound (EUS) now permit detailed morphologic assessment complemented by cyst-fluid biochemical markers, proteomic signatures, and comprehensive genomic profiling using next-generation sequencing. Parallel progress in artificial intelligence (AI) further strengthens diagnostic precision by integrating EUS features with multimodal biomarker data to reduce subjectivity and support individualized clinical decision-making. This review introduces an EUS-based multimodal diagnostic framework of PCLs that integrates morphological evaluation, cyst-fluid biochemical testing, molecular profiling, and AI-assisted analysis. By synthesizing current evidence, we outline how the integrative approach enhances diagnostic accuracy, biological interpretability, and individualized risk stratification for PCLs.

## 1. Background

Pancreatic cystic lesions (PCLs) are increasingly identified in clinical practice, largely due to the widespread use of high-resolution cross-sectional imaging and the aging population. Recent imaging-based studies indicate that the prevalence of PCLs ranges from approximately 10–20% in the general population and increases substantially with age, exceeding 20% in elderly individuals undergoing abdominal imaging. Although many PCLs are incidentally detected and remain clinically indolent, a significant subset represents precursor lesions to pancreatic ductal adenocarcinoma, particularly mucinous neoplasms capable of progressing to high-grade dysplasia or invasive malignancy [1,2,3]. Given the marked biological heterogeneity—from benign cysts to premalignant and malignant lesions—accurate classification remains challenging. Misclassification has important clinical consequences, as underestimation of high-risk lesions may delay appropriate intervention, whereas overestimation of benign cysts may lead to unnecessary surgical resection and prolonged surveillance. Contemporary studies further demonstrate that cross-sectional imaging alone frequently fails to reliably distinguish mucinous from non-mucinous cysts, highlighting the clinical impact of diagnostic inaccuracy. Therefore, precise assessment of cyst type and biological risk at the time of detection is essential for guiding surveillance strategies and therapeutic decision-making.

Despite advances in computed tomography (CT) and magnetic resonance imaging (MRI), non-invasive characterization of PCLs remains challenging. Overlapping morphological features—such as septations, mural nodules, wall thickening, and ductal communication—limit the ability of cross-sectional imaging to reliably distinguish mucinous from non-mucinous lesions. Contemporary studies report that diagnostic accuracy remains limited, with correct classification achieved in approximately 50–70% of cases and falling below 50% in some studies [4,5]. Furthermore, cyst-fluid cytology obtained through fine-needle aspiration provides supplementary diagnostic information but is inherently limited by low cellularity, leading to suboptimal sensitivity and frequent false-negative results for high-grade dysplasia or malignancy [6]. In addition, variability among international management guidelines, including the American Gastroenterological Association (AGA) and the Fukuoka consensus, reflects inconsistencies in recommendations for surgical intervention and surveillance, with AGA guidelines adopting more conservative thresholds, whereas Fukuoka criteria favor earlier surgical consideration [5]. Collectively, these limitations highlight the need for more refined diagnostic strategies capable of integrating multiple dimensions of information.

Endoscopic ultrasound (EUS) has emerged as a pivotal modality for the evaluation of PCLs. Its superior spatial resolution allows detailed assessment of cyst architecture, including mural nodules, septations, and ductal communication, thereby improving lesion characterization beyond cross-sectional imaging alone [7,8,9]. Moreover, EUS uniquely enables the acquisition of cyst fluid or targeted tissue for biochemical, cytologic, and molecular analyses, providing critical information on neoplastic potential, mucinous differentiation, and dysplasia [10,11,12]. These capabilities establish EUS as a central platform for comprehensive evaluation of PCLs, with assessment evolving from morphological characterization to a multimodal approach incorporating cyst-fluid biomarkers and molecular profiling. Accordingly, the increasing complexity of these multidimensional data has, in turn, driven the integration of artificial intelligence (AI) to facilitate more objective and efficient diagnostic analysis [13,14,15,16,17,18]. Building on this foundation, we propose a multimodal diagnostic framework aimed at improving diagnostic precision, reducing unnecessary interventions, and enabling individualized management of PCLs.

## 2. Overview of Pancreatic Cystic Lesions

PCLs comprise a heterogeneous set of entities with distinct epithelial origins, clinical behavior, and malignant potential. They are broadly classified into non-neoplastic and neoplastic lesions [14]. Non-neoplastic cysts are predominantly pseudocysts, which lack an epithelial lining and typically occur after acute or chronic pancreatitis or pancreatic ductal disruption [15,16]. Although usually benign, pseudocysts may resemble true neoplasms on imaging and warrant differentiation when the clinical history is unclear. Neoplastic cysts, by contrast, are epithelial lesions with variable risk of malignant transformation. They are subdivided into mucinous and non-mucinous neoplasms. Mucinous cystic neoplasms (MCNs) and intraductal papillary mucinous neoplasms (IPMNs) represent the principal mucin-producing lesions. MCNs are characterized by ovarian-type stroma and occur almost exclusively in women, typically within the pancreatic body or tail. IPMNs arise from the main pancreatic duct and/or its side branches and are classified into main-duct, branch-duct, and mixed-type subtypes based on ductal involvement. Main-duct and mixed-type IPMNs carry the highest likelihood of harboring high-grade dysplasia or invasive carcinoma, whereas most branch-duct IPMNs follow a more indolent course but still warrant surveillance due to variable progression risk [17,18,19]. Non-mucinous neoplastic cysts include serous cystic neoplasms (SCNs), solid pseudopapillary neoplasms (SPNs), and cystic neuroendocrine tumors (cNETs). SCNs demonstrate characteristic microcystic architecture and are overwhelmingly benign, although differentiation from mucinous cysts is often required to avoid unnecessary surgery. SPNs occur predominantly in young women and, despite being low-grade malignancies, require accurate identification because surgical resection is typically curative. cNETs and cystic degeneration of pancreatic ductal adenocarcinoma comprise a smaller but clinically important subset in which imaging alone may be insufficient for reliable diagnosis [20,21,22].

Although CT and MRI remain foundational in identifying PCLs, their diagnostic accuracy is limited by overlapping morphological features. For example, mural nodules, septations, wall thickening, and ductal communication may appear in both benign and premalignant lesions, reducing the reliability of purely morphology-based classification. Recent studies indicate that cross-sectional imaging alone misclassifies a substantial proportion of mucinous and high-risk cysts, reinforcing the need for higher-resolution, biologically informed assessment modalities [23,24]. These diagnostic limitations underscore the increasingly central role of EUS, which enables detailed evaluation of internal cystic structure while facilitating cyst-fluid and tissue acquisition for biochemical, cytologic, and molecular profiling.

## 3. Comprehensive EUS Evaluation of Pancreatic Cystic Lesions

### 3.1. Morphological Evaluation of PCLs

EUS provides high-resolution morphological assessment of pancreatic cystic lesions (PCLs), enabling detailed evaluation of cyst size, wall characteristics, internal septations, mural nodules, solid components, and communication with the main pancreatic duct. These parameters constitute the structural foundation for initial lesion characterization and are integral to guideline-based risk stratification frameworks. Mural nodules and solid components represent critical morphological indicators, as their presence is closely associated with high-grade dysplasia and invasive malignancy, particularly in IPMNs. Main pancreatic duct dilation and cyst–duct communication constitute typical morphological features of IPMNs and play a central role in diagnostic classification and risk stratification [14]. Additional parameters, including cyst size and wall thickening, are incorporated into risk assessment frameworks and contribute to the evaluation of malignant potential. EUS morphological evaluation additionally contributes to cyst subtype differentiation. Ductal communication is indicative of IPMNs, whereas a thick-walled cyst with internal septations is suggestive of MCNs, and a microcystic or honeycomb architecture with thin septa is characteristic of SCNs. However, substantial overlap in morphological features across cyst subtypes limits the specificity of EUS-based assessment. Morphology alone provides limited discrimination between mucinous and non-mucinous lesions and is insufficient for the reliable detection of early malignant transformation [25]. Accordingly, morphological assessment should be integrated with functional imaging and EUS-guided tissue acquisition to improve diagnostic accuracy and risk stratification.

### 3.2. Functional Evaluation of PCLs

Recent advances in EUS technology have enabled the functional evaluation of PCLs, primarily through contrast-enhanced EUS (CE-EUS) and EUS elastography, which provide complementary information beyond conventional morphological assessment. CE-EUS facilitates the assessment of microvascular perfusion within cyst walls, septa, and mural nodules, thereby improving the characterization of intracystic structures. Particularly, enhancing mural nodules or vascularized solid components are indicative of neoplastic tissue, whereas non-enhancing intra-cystic components are more frequently observed in benign or non-neoplastic lesions [26]. By enabling the distinction between vascularized and avascular intracystic components, CE-EUS contributes to more accurate identification of lesions with increased malignant potential. EUS elastography provides adjunctive information through the evaluation of tissue stiffness within cyst walls, mural nodules, and adjacent tissue. Increased stiffness is generally associated with fibrotic or neoplastic components, whereas softer patterns are more commonly observed in non-neoplastic lesions. However, limited standardization and variability in measurement techniques restrict the role of elastography to a complementary modality in the assessment of PCLs [27,28]. Combined morphological and functional EUS assessments can enable more accurate differentiation between low- and high-risk lesions prior to EUS-guided sampling.

### 3.3. EUS-Guided Tissue and Fluid Acquisition

Although morphologic and functional EUS assessment provides essential information for characterizing PCLs, imaging alone often lacks the ability to determine epithelial phenotype, mucinous differentiation, or the degree of dysplasia. EUS-guided cystic fluid and tissue acquisition therefore represents a critical extension of cyst evaluation, bridging structural assessment with downstream cytologic, biochemical, and molecular analysis [29,30].

EUS-guided fine-needle aspiration (EUS-FNA) remains a widely used technique for cyst fluid acquisition and evaluation. Cyst fluid obtained via EUS-FNA can be subjected to multiparametric analysis at the physical, biochemical, and molecular levels, thereby providing key information for lesion classification and risk assessment [31,32,33]. The string sign test is a direct bedside method for assessing viscosity. During the operation, if the fluid-filled sac is stretched between the fingers to form a string ≥ 1 cm in length, it can be judged as positive, indicating a mucinous cyst (MCNs or IPMNs). This test reflects the mucin content of the cystic fluid and demonstrates high specificity (up to 95%) and positive predictive value (94%), supporting its utility as a rapid technique for identifying mucinous lesions. However, the test is inherently operator-dependent, as the applied stretching force may vary among operators [34,35,36]. The cytological analysis of the fluid obtained by EUS-FNA may improve the diagnostic accuracy of SPNs, but the diagnostic sensitivity is low [37]. Even in the absence of malignant cells on cytology, malignancy cannot be definitively excluded due to limited cellularity.

To overcome these challenges, EUS-guided fine-needle biopsy (EUS-FNB) and EUS through-the-needle micro-forceps biopsy (EUS-TTNB) have emerged as superior sampling modalities. Both techniques provide histological architecture rather than isolated cell fragments, significantly increasing diagnostic yield. Recent prospective and multicenter studies demonstrated that EUS-TTNB can yield diagnostic histology in 70–90% of sampled lesions, outperforming FNA for identifying mucinous epithelium, ovarian-type stroma in MCNs, and dysplasia in IPMNs [38,39,40]. Two independent comparative studies further show that FNB needles (e.g., Franseen or fork-tip designs) improve tissue yield and diagnostic accuracy for cNETs, solid pseudopapillary neoplasms, and cystic degeneration of ductal adenocarcinoma—pathologies in which architectural integrity is pivotal for classification [41,42].

EUS-guided sampling is particularly valuable when morphological features remain equivocal, even after advanced functional imaging. For example, targeted sampling of mural nodules identified on CE-EUS enables differentiation between vascularized neoplastic nodules and avascular mucin globules, thereby refining the assessment of dysplasia and reducing the risk of misclassification. In addition, EUS-TTNB has demonstrated utility in cysts with indeterminate imaging characteristics, such as atypical septations or thickened walls, improving diagnostic accuracy. Importantly, EUS-guided sampling provides the procedural basis for subsequent biochemical, cytologic, and molecular analyses. The diagnostic yield of these downstream modalities is closely dependent on the quality and quantity of specimens obtained. Taken together, these EUS-based techniques provide complementary information for the evaluation of PCLs. The principal EUS-based morphological and functional approaches for PCLs evaluation are summarized in Table 1.

## 4. Molecular Profiling and Biomarker Characterization of Pancreatic Cystic Lesions

Cyst fluid obtained via EUS sampling, and less commonly during surgical resection, serves as a valuable substrate for PCLs associated biomarker testing. These biomarkers can reflect epithelial secretion, ductal communication, inflammatory signaling, and underlying genetic alterations [43,44]. Based on their biological characteristics and clinical applicability, cyst-related biomarkers can be broadly categorized into three groups: conventional biochemical markers, cyst-fluid biomarkers with potential clinical utility and emerging genomic/molecular testing. The key biomarker features of major PCLs are summarized in Table 2.

### 4.1. Conventional Biochemical Biomarkers

These include carcinoembryonic antigen (CEA), glucose, and amylase, which remain fundamental for initial cyst characterization. They are simple, reproducible, and widely available, representing the earliest biochemical basis for distinguishing mucinous from non-mucinous cysts.

#### 4.1.1. CEA

CEA is the most widely used and well-validated biochemical marker for differentiating mucinous from non-mucinous pancreatic cysts. A commonly used cutoff is approximately 192 ng/mL, providing a specificity of 82–92% and a sensitivity of 45–71%. However, CEA cannot distinguish benign from malignant mucinous lesions. Recent guidelines continue to endorse cyst-fluid CEA as a primary discriminator, although they also recommend integrating it with additional parameters to improve reliability [13,29].

#### 4.1.2. Cyst-Fluid Glucose

Cyst-fluid glucose has recently emerged as a low-cost and reliable discriminator, showing accuracy comparable to or exceeding that of CEA in several prospective studies. The test requires minimal fluid volume and can be rapidly performed with bedside glucometers during EUS-FNA. Glucose levels in mucinous pancreatic cysts are usually below 50 mg/dL, a cutoff that provides a specificity of about 89% (83–95%) and a sensitivity of 93% (89–97%). The combination assessment of glucose and CEA is particularly useful in cases with indeterminate CEA values [45,46,47].

#### 4.1.3. Cyst-Fluid Amylase

Another useful cyst fluid marker is amylase: Cyst-fluid amylase reflects ductal communication rather than epithelial neoplastic potential. A high level of amylase may indicate a pseudocyst or IPMN. When the concentration is below 250 U/L, a pseudocyst can be effectively ruled out, and it is highly specific (although the sensitivity is relatively low at 44%) [15].

### 4.2. Cyst-Fluid Biomarkers with Potential Clinical Application Value

#### 4.2.1. Mucin-Related Glycoproteins

MUC1 is highly expressed in most malignant mucinous cysts. SCNs are mainly manifested as cystic formations, but sometimes they are present as solid lesions. In such cases, immunohistochemical testing is helpful for differential diagnosis. Neuron-specific enolase, α-inhibin and mucin-related glycoprotein (MUC1 and MUC6) are specific immune profiles for SCNs [48].

#### 4.2.2. CA19-9

Among tumor markers evaluated in PCLs, CA19-9 is the most extensively studied biomarker after CEA. Elevated serum or cyst fluid CA19-9 levels are often associated with malignant or high-grade dysplastic cystic neoplasms, particularly IPMNs and MCNs. Several studies have demonstrated that CA19-9 exhibits moderate diagnostic performance in distinguishing benign from malignant cystic lesions, with reported sensitivity and specificity values around 72% and 75%, respectively, when cyst fluid concentrations exceed approximately 364.55 ng/mL [25,49]. However, the diagnostic utility of CA19-9 remains limited due to its relatively low sensitivity and potential elevation in non-malignant pancreatic diseases or obstructive jaundice. Its specificity for differentiating mucinous from non-mucinous cysts is also inferior to that of CEA [50,51]. Consequently, current guidelines and reviews recommend that CA19-9 be used as an adjunctive marker, rather than a standalone diagnostic tool, ideally interpreted in conjunction with cyst morphology, cytology, and biochemical markers such as CEA.

#### 4.2.3. VEGF-A

Vascular endothelial growth factor (VEGF), particularly VEGF-A, has emerged as a highly accurate biomarker in distinguishing SCNs from other PCLs. Carr et al. demonstrated that VEGF-A levels in pancreatic cyst fluid are markedly elevated in SCNs compared to all other cyst types (median 35,598 pg/mL vs. 2149 pg/mL, *p* < 0.0001). A VEGF-A threshold of >5000 pg/mL achieved 100% sensitivity and 83.7% specificity for identifying SCNs, and when combined with a CEA level ≤ 10 ng/mL, diagnostic performance improved to 95.5% sensitivity and 100% specificity, approaching the accuracy of pathological diagnosis [52,53].

### 4.3. Emerging Genetic Molecular Testing

Molecular and genomic profiling has recently become an integral component in the diagnostic evaluation of PCLs. Unlike biochemical or protein-based markers that reflect secretory and metabolic activity, molecular biomarkers directly characterize the underlying genomic alterations. The application of next-generation sequencing (NGS) to cyst-fluid samples has markedly enhanced the sensitivity, specificity, and breadth of preoperative cyst characterization [54].

#### 4.3.1. Diagnostic Mutations for Cyst Typing

KRAS is the “initiating mutation” of mucinous cysts (IPMNs, MCNs), with a positive rate of approximately 60–70% in IPMNs and about 40% in MCNs. It has a specificity of up to 95–100% for differentiating mucinous from non-mucinous lesions, but its sensitivity remains relatively low (47–67%) [55]. GNAS mutations are mainly found in IPMNs (31–40%) and are almost negative in MCNs. Its specificity is close to 100%, and combined with KRAS detection, the diagnostic specificity of mucinous cysts can be increased to nearly 100% [56]. Progression mutations such as TP53, SMAD4, CDKN2A, and PIK3CA usually occur at the high-grade dysplasia (HGD) or invasive cancer (IC) stage. Although the sensitivity is relatively low (9–42%), the specificity is extremely high (92–98%), and can be used as an important molecular marker for the risk of malignancy [2]. Based on the multi-gene combined analysis of NGS, the diagnostic efficacy has been further improved: the combined detection of KRAS/GNAS and TP53, PIK3CA, PTEN, etc., has a sensitivity of up to 89% for mucinous lesions and a specificity of 100%, and has a sensitivity of about 88–89% for HGD [57].

#### 4.3.2. NGS-Based Platforms

NGS-based diagnostic platforms have substantially improved the molecular characterization and clinical triage of PCLs. The PancreaSeq system, including its updated DNA/RNA-based Genomic Classifier (PancreaSeq GC), integrates mutations (KRAS, GNAS, RNF43, VHL, TP53, SMAD4, PIK3CA, PTEN), gene fusions, copy-number alterations, and RNA expression data. This platform can accurately classify cyst types and identify advanced neoplasia. PancreaSeq GC achieved 95% sensitivity and 100% specificity for mucinous precursor lesions and 82% sensitivity/100% specificity for advanced neoplasia, outperforming guideline-based criteria. In a prospective multi-institutional cohort, PancreaSeq GC also demonstrated high accuracy for mucinous cysts (MAPK/GNAS mutations: 90% sensitivity/100% specificity) and for detecting high-grade dysplasia using TP53/SMAD4/MTOR-pathway alterations, with performance superior to AGA and Fukuoka guidelines [58,59].

#### 4.3.3. Liquid-Biopsy Markers

Recent studies have also explored cell-free DNA (cfDNA) and microRNA (miRNA) signatures as noninvasive molecular indicators of neoplastic potential. cfDNA concentrations are significantly higher in IPMNs, improving detection of key mutations such as GNAS R201C/H, which show strong diagnostic specificity (71%) for mucinous cysts. Epigenetic cfDNA markers remain less well characterized but represent an emerging area of interest [60]. MicroRNA profiling shows clearer value: a validated cyst-fluid panel (miR-31-5p, miR-483-5p, miR-99a-5p, miR-375) accurately distinguishes serous from mucinous or malignant lesions, with high sensitivity and specificity; additional miRNAs (miR-200 family, miR-224, miR-363) help differentiate IPMN subtypes and grades [61].

## 5. Artificial Intelligence in the Assessment of PCLs

### 5.1. Imaging-Based Artificial Intelligence for PCL Evaluation

EUS remains the cornerstone for the diagnostic evaluation of PCLs because of its superior spatial resolution and ability to visualize cyst morphology, mural nodules, and ductal communication. However, the interpretation of EUS images is inherently operator-dependent, subject to interobserver variability and qualitative bias. The integration of AI into EUS imaging aims to overcome these limitations by facilitating objective, reproducible image interpretation through quantitative pattern recognition and automated feature extraction [62,63]. Recent studies have demonstrated that convolutional neural networks (CNNs) and radiomics-based models can distinguish PCL subtypes and identify high-risk lesions with diagnostic accuracy comparable to experienced EUS operators. Leang et al. reported that a CNN trained on EUS images achieved an area under the curve (AUC) of 0.93 for differentiating IPMNs from mucinous cystic neoplasms MCNs [64].

Similarly, Yashika et al. applied radiomics feature analysis to CE-EUS, achieving 90% sensitivity in identifying mural nodules suggestive of high-grade dysplasia [65]. Beyond classification, AI algorithms have shown potential for automated quantification of cyst wall irregularity, septation, and main pancreatic duct dilation—features strongly correlated with malignant transformation risk. Complementary applications have also emerged in EUS elastography and contrast-enhanced harmonic imaging, where AI aids in differentiating cystic from solid components and assessing tissue stiffness. In a retrospective study, Lee et al. developed a deep learning model incorporating both B-mode and elastography signals, yielding improved discrimination of high-risk IPMNs compared with standard image review [66]. These advancements illustrate the promise of multimodal EUS-AI systems capable of integrating multiple imaging parameters to achieve comprehensive lesion assessment.

While EUS remains the principal imaging modality for PCLs, similar AI frameworks have been explored for CT and MRI, primarily in radiomics-based risk prediction. Cui et al. utilized MRI radiomic signatures combined with clinical features to predict high-grade dysplasia in IPMNs with an AUC of 0.92, surpassing traditional morphological criteria [67]. Such cross-modality models highlight the potential of AI to unify EUS and cross-sectional imaging data into a cohesive, quantitative diagnostic platform.

Despite these advances, significant challenges persist before AI can be routinely integrated into clinical EUS practice. Model performance is often limited by small, single-center datasets and heterogeneous image acquisition parameters. The lack of external validation and standardized image repositories restricts generalizability. Moreover, explainability remains a concern; most deep learning models function as “black boxes,” offering limited insight into decision pathways. Emerging research into explainable AI (XAI) using Grad-CAM or SHAP-based visualization may enhance clinical reliability and transparency [68,69,70].

In summary, imaging-based AI, particularly within EUS, has demonstrated substantial potential in refining PCL classification, identifying high-risk features, and reducing diagnostic subjectivity. As multi-institutional datasets and federated learning frameworks mature, EUS-AI models are expected to evolve from proof-of-concept systems into validated, clinically deployable tools, forming a critical component of precision diagnostics for PCLs.

### 5.2. AI-Assisted Analysis of Biomarkers and Molecular Profiles in PCLs

#### 5.2.1. AI in Biochemical Biomarker Evaluation

CEA, glucose, and amylase are widely analyzed in pancreatic cyst fluid obtained via EUS-FNA to differentiate between mucinous and non-mucinous cystic lesions. However, the diagnostic utility of these biochemical markers is often limited due to significant overlap in biomarker levels across different cyst types. AI provides a valuable tool to address this limitation by integrating multiple biochemical markers to improve diagnostic accuracy. Machine learning (ML) models, particularly random forests and support vector machines (SVM), have been employed to analyze multi-biomarker data. These AI-based models can identify complex, non-linear patterns that are not easily discernible by traditional statistical methods [71]. Huang et al. demonstrated that an AI model integrating CEA, glucose, and amylase achieved an AUC of 0.90 for distinguishing MCNs from SCNs [72]. These findings suggest that AI can enhance the diagnostic performance of biochemical markers, offering more reliable and objective risk stratification for PCLs.

#### 5.2.2. AI in Emerging Molecular and Genomic Biomarker Analysis

NGS has led to significant advances in the molecular analysis of PCLs, revealing important genomic alterations such as mutations in KRAS, GNAS, TP53, and SMAD4, which are particularly associated with mucinous cysts and higher malignancy potential. The integration of AI with genomic data allows for better interpretation and risk prediction in PCLs by combining genetic mutations with clinical and imaging features.

AI has emerged as a key tool in the analysis of emerging molecular biomarkers, including genetic mutations and epigenetic changes, which are critical in PCL characterization. Models that combine genomic data, such as those used in CompCyst and PancreaSeq-ML, have significantly improved diagnostic accuracy. For example, the CompCyst model uses machine learning to integrate molecular, clinical, and imaging data, which has helped reduce unnecessary surgeries while maintaining high sensitivity for identifying high-risk cysts. Similarly, PancreaSeq-ML integrates DNA and RNA sequencing data with AI to achieve an AUC of 0.95 in distinguishing high-risk IPMNs from benign cysts [73].

In addition to genomic markers like KRAS and GNAS, proteomic profiling has been used to identify biomarkers that may indicate malignant transformation. AI models have been applied to proteomic data to identify patterns that would be difficult for clinicians to detect manually. Studies by Yoon et al. have demonstrated that AI can analyze proteomic profiles to predict malignant transformation with high diagnostic accuracy [74]. This integration of emerging molecular data with AI may transform how PCLs are assessed, offering more precise and individualized risk stratification.

## 6. Multi-Model Approach for Risk Stratification and Personalized Evaluation of PCLs

The inherent heterogeneity of PCLs, together with the substantial overlap in their imaging features, underscores the limitations of single-modality diagnostic approaches. As clinical practice advances toward biologically informed and precision-oriented evaluation, a comprehensive multimodal framework has emerged to address these challenges. Anchored in EUS and enriched by biochemical, molecular, and computational data, this framework enables a more integrated characterization of PCLs. Rather than representing a simple aggregation of diagnostic tools, this approach defines a coordinated architecture in which each modality contributes complementary and non-redundant information. Within this paradigm, EUS functions as the central platform for data acquisition and integration, providing high-resolution morphological and functional assessment while facilitating targeted tissue and fluid sampling. By linking structural imaging with downstream biological and computational analyses, this multimodal strategy shifts PCs evaluation from morphology-based classification toward a more comprehensive, biology-informed diagnostic paradigm, ultimately supporting more precise and individualized risk stratification.

Building on this integrated framework, molecular and genomic profiling further enhances diagnostic resolution by identifying lineage-specific mutations and alterations associated with high-grade dysplasia. These molecular signatures not only refine histological prediction but also provide mechanistic insights into cyst behavior and malignant potential. The increasing availability of NGS platforms enables the simultaneous assessment of multiple genomic pathways, allowing a more comprehensive evaluation of cyst evolution. Within this multidimensional data landscape, AI approaches enable the integrative analysis of heterogeneous inputs. Machine-learning models can extract quantitative features from EUS-derived imaging, model complex biomarker interactions, and identify genomic patterns associated with disease progression. By facilitating the synthesis of these complementary data sources, AI may reduce interpretative variability, improve reproducibility, and support probabilistic risk stratification. This integrative strategy is particularly valuable in lesions with indeterminate or overlapping features, where conventional assessment may be insufficient to provide clear diagnostic or risk stratification guidance.

The clinical relevance of this multimodal approach is best illustrated through representative practice-oriented scenarios. In patients with incidentally detected PCLs and indeterminate findings on cross-sectional imaging, integrated EUS-based evaluation combined with cyst-fluid biomarkers improves diagnostic confidence in distinguishing mucinous from non-mucinous lesions, thereby reducing unnecessary surgical intervention and optimizing surveillance strategies [75,76]. This distinction is particularly important in small, asymptomatic cysts, where overtreatment may result in avoidable morbidity. In lesions with suspected high-risk features, such as mural nodules or main pancreatic duct dilation, advanced EUS-based assessment can further refine risk stratification by clarifying the nature of intracystic structures and vascular patterns, thereby influencing decisions regarding surgical referral versus continued surveillance [77,78]. During longitudinal follow-up, when cysts exhibit interval growth or evolving morphology, repeated integration of EUS findings with biochemical and molecular data enables dynamic reassessment of malignancy risk, supporting a shift from static classification toward adaptive, biology-informed evaluation. In cases where morphological and functional assessment remains inconclusive, EUS-guided tissue acquisition provides additional diagnostic information. The incorporation of histological and molecular data can improve lesion classification and support individualized risk assessment, particularly in complex or atypical cysts where conventional pathways are insufficient [79,80]. Collectively, these scenarios demonstrate how multimodal integration enhances diagnostic precision, reduces uncertainty, and supports more rational, patient-specific decision-making across different stages of PCL evaluation.

A central innovation of the multimodal paradigm is the incorporation of AI-driven analysis systems capable of integrating these heterogeneous inputs. Machine learning models can quantify subtle EUS-derived textural patterns, interpret complex biomarker interactions, and classify genomic trajectories indicative of progression. By synthesizing multimodal signals, AI reduces subjectivity, improves reproducibility, and supports probabilistic risk stratification that surpasses traditional rule-based criteria. This integration is especially valuable for lesions with equivocal features, where conventional assessments often struggle to provide actionable clarity.

Beyond enhancing initial diagnostic accuracy, the multimodal framework also introduces a crucial capacity for dynamic and individualized longitudinal assessment. As cyst morphology and biological markers evolve over time, updated EUS findings, repeated biochemical measurements, and new genomic data can be assimilated to refine malignancy risk in real time. This temporal adaptability represents a significant advancement over static guideline-based management, enabling surveillance intervals and intervention thresholds to be personalized based on evolving biological risk rather than fixed morphologic criteria.

Despite these advances, the implementation of EUS-based multimodal evaluation remains constrained by operator variability, which can affect image acquisition, interpretation, and sampling performance. Studies have reported only moderate interobserver agreement for key EUS features, including mural nodules and ductal communication, underscoring limitations in reproducibility. In addition, variability in sampling techniques—such as needle selection, targeting strategy, and specimen handling—may further influence diagnostic yield and downstream analysis. To address these challenges, comprehensive strategies for standardization are required. Structured training programs and competency-based assessment can improve the consistency of EUS performance. In addition, the use of consensus-based criteria for interpreting high-risk features—such as those defined in the Fukuoka and AGA guidelines—may enhance interobserver agreement. The implementation of standardized reporting systems further facilitates uniform documentation and clinical communication. In parallel, emerging AI-assisted approaches provide an additional pathway toward standardization. Machine learning models applied to EUS imaging have demonstrated the ability to extract quantitative features, support lesion classification, and reduce subjective interpretation. By enabling objective analysis and integration of multimodal data, AI may enhance reproducibility and support more consistent decision-making within a standardized diagnostic framework.

**Figure 1:**
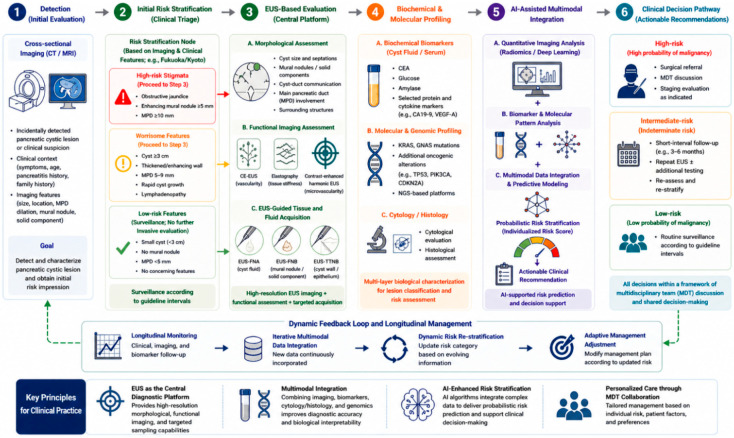
EUS-Anchored Multimodal Diagnostic Roadmap for Pancreatic Cystic Lesions. Abbreviations: CE-EUS, contrast-enhanced endoscopic ultrasound; CT, computed tomography; EUS, endoscopic ultrasound; FNA, fine-needle aspiration; FNB, fine-needle biopsy; TTNB, through-the-needle biopsy; MPD, main pancreatic duct; MDT, multidisciplinary team; NGS, next-generation sequencing.

Taken together, the multimodal framework defines a unified diagnostic ecosystem that integrates EUS-based imaging, molecular profiling, and AI-assisted analysis into a coherent pathway. By combining complementary data streams, this approach improves diagnostic precision and enables more informed patient stratification for surgery or surveillance. It further provides a scalable platform for incorporating emerging biomarkers and analytical technologies, reflecting a shift toward biology-informed, precision evaluation of PCLs with potential to improve clinical outcomes (Figure 1).

## 7. Summary and Future Directions

The proposed multimodal framework represents a conceptual evolution in the evaluation of PCLs, integrating EUS-based comprehensive assessment, biochemical and molecular profiling, and AI-assisted analysis into a unified diagnostic workflow. By enabling the synthesis of structural, biological, and computational data, this approach supports individualized interpretation of cyst behavior, overcomes the limitations of morphology-based assessment, and improves the precision of malignancy risk stratification. Emerging evidence indicates that the integration of EUS-guided cyst-fluid analysis with NGS and computational modeling enhances diagnostic accuracy, reduces unnecessary surgical interventions, and enables dynamic monitoring of PCL malignant progression during surveillance [81,82].

Despite these advances, the multimodal framework remains subject to several limitations. Variability in imaging acquisition, biomarker assay protocols, and molecular profiling workflows compromises reproducibility across institutions. In parallel, the reliance of current AI models on retrospective or relatively small datasets constrains generalizability. The limited interpretability of deep learning algorithms further hinders clinical adoption, given the insufficient transparency of decision-making processes. Moreover, advanced molecular approaches, including NGS and proteomic analyses, remain resource-intensive and are not universally accessible, thereby contributing to disparities in implementation across healthcare systems [83,84].

Future progress will depend on addressing these challenges through coordinated efforts in data standardization, technological innovation, and clinical translation. The establishment of unified standards for EUS acquisition, cyst-fluid biomarker characterization, and genomic sequencing workflows is essential to reduce inter-institutional variability and improve external validity. The development of standardized reporting systems, shared reference datasets, and multicenter prospective cohorts—potentially enabled through privacy-preserving data-sharing frameworks—will be critical for building robust and generalizable diagnostic models.

To improve accessibility and scalability, future strategies should prioritize cost-effective diagnostic solutions, including streamlined biomarker panels with validated clinical utility and simplified sequencing approaches. In parallel, the deployment of cloud-based or embedded AI decision-support systems may facilitate real-time data integration and standardized interpretation across diverse clinical settings. Prospective validation studies and cost-effectiveness analyses will be essential to guide clinical adoption, inform reimbursement strategies, and define appropriate use thresholds.

**Figure 2:**
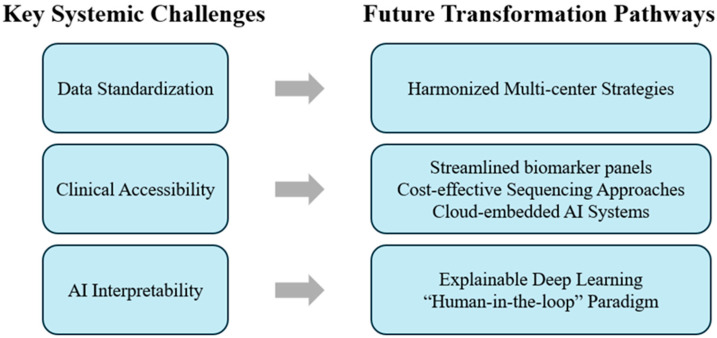
Key systemic challenges and future transformation pathways for EUS-based, multi-modal approach for evaluation of PCLs: This schematic summarizes the major barriers limiting the implementation of EUS-based multimodal frameworks, variability in data acquisition and annotation (data standardization), uneven availability of advanced biomarker and molecular platforms (clinical accessibility), and the limited transparency of current AI-driven analysis models (AI interpretability). Corresponding future directions include harmonized multicenter strategies to unify imaging and molecular data standards, federated AI collaborations to enable secure inter-institutional model training, and the development of explainable deep learning architectures to enhance model transparency and clinical acceptance.

Addressing the interpretability of AI models represents another key priority. The integration of explainable AI (XAI) approaches—such as feature attribution, uncertainty quantification, and model-agnostic interpretability frameworks—may enhance transparency and align algorithmic outputs with clinical reasoning. The development of standardized validation protocols, reporting guidelines, and clinician–AI interaction frameworks will further support safe and effective implementation. In the long term, a “human-in-the-loop” paradigm, in which AI augments rather than replaces expert judgment, is likely to provide the most practical and clinically acceptable model for PCL risk assessment (Figure 2).

Collectively, the future of PCL evaluation lies in the convergence of standardized data acquisition, scalable implementation pathways, and interpretable AI systems. As these advances mature and undergo rigorous validation, they have the potential to transform PCL risk assessment into a dynamically adaptive, biologically informed, and clinically actionable process.

## Figures and Tables

**Table 1:** EUS Morphological and Functional Characteristics of Major Pancreatic Cystic Lesions and their Diagnostic and Risk-Stratification Value. MPD: Main pancreatic duct.

Technique	Target Parameter	Imaging and Sampling Findings	Diagnostic and Risk-Stratification Value	Strengths	Limitations
Conventional EUS morphology	Cyst structure; ductal anatomy	Multilocularity; wall thickening; mural nodules; MPD dilation; ductal communication	Lesion characterization; identification of worrisome features (e.g., mural nodules, ductal dilation)	High-resolution imaging; real-time assessment	Operator dependence; limited specificity in overlapping features
CE-EUS	Vascularity of mural nodules and cyst wall	Hyper-enhancing mural nodules; non-enhancing intracystic material	Differentiation of vascularized neoplastic tissue from avascular intracystic content	Microvascular visualization; contrast-enhanced characterization	Requirement for contrast agents; operator dependence
EUS elastography	Tissue stiffness	Increased stiffness (hard pattern); soft pattern	Adjunctive assessment of tissue composition (fibrotic or neoplastic vs. non-neoplastic)	Noninvasive stiffness assessment; functional tissue characterization	Limited standardization; adjunctive diagnostic role
EUS-FNA	Cyst fluid	Low cellularity aspirate; fluid biomarkers	Mucinous cyst identification; cytologic confirmation of malignancy when positive	Wide availability; fluid-based biochemical and molecular analysis	Low cellularity; limited sensitivity; false-negative cytology
EUS-FNB	Cyst wall; mural nodules; solid components	Core tissue with preserved architecture	Histologic diagnosis in lesions with mural nodules or solid components	Core tissue acquisition; preservation of histologic architecture	Limited utility in purely cystic lesions; technical dependence
EUS-TTNB	Intracystic wall; septa; epithelium	Direct epithelial tissue sampling	Histologic characterization of cyst epithelium; cyst subtype classification	Direct epithelial sampling; improved histologic yield	Procedure-related adverse events; technical complexity

**Table 2:** Clinical, Imaging, and Molecular Characteristics of Major Pancreatic Cystic Lesions.

Features	BD-IPMNs	MD-IPMNs or MT-IPMNs	MCNs	SCNs	cNETs	SPNs
Age (years)	50–70	50–70	30–50	60–80	50–60	20–40
Gender	F = M	F = M	F (>95%)	F (70%)	M > F	F (>80%)
Location in pancreas	50% in head and uncinate	Any	90% in body and tail	Any	Any	Any
Clinical symptoms	Mostly no symptom when lesions are small	Mostly no symptom when lesions are small	Up to 50% of cases (might associate with compressive symptoms)	Up to 50% of cases (might associate compressive symptoms)	90% asymptomatic. Functional symptoms (depend on secreted hormone)	Mostly no. Abdominal pain or discomfort (37%)
Calcification	No	No	Rare peripheral calcification	Central calcification in 30–40%	No	Irregular
Malignant potential	Yes	Yes	Yes	None	Irregular	Irregular
Glucose	Low	Low	Low	Normal	-	-
Amylase (U/L)	>250	>250	<250	<250	-	>250
CEA (ng/mL)	>192	>192	>192	<5	<5	<5
KRAS mutation	Yes	Yes	Yes	Very rare		None
GNAS mutation	-	Yes	Yes	-	-	-

This table summarizes the clinical, imaging, and molecular characteristics of major pancreatic cystic lesions and supports differential diagnosis based on imaging patterns, morphologic features on EUS, biochemical markers, and key genomic alterations. (Abbreviations: BD-IPMNs: Branch-duct intraductal papillary mucinous neoplasms; MD-IPMNs: Main-duct intraductal papillary mucinous neoplasms; MT-IPMNs: Mixed-type intraductal papillary mucinous neoplasms; MCNs: Mucinous cystic neoplasms; SCNs: Serous cystic neoplasms; cNETs: Cystic neuroendocrine tumors; SPNs: Solid pseudopapillary neoplasms; CEA: Carcinoembryonic antigen; KRAS (Kirsten Rat Sarcoma Viral Oncogene Homolog); GNAS (Guanine Nucleotide-Binding Protein GNAS Complex Locus).

## Data Availability

No new data were created or analyzed in this review.

## Abbreviations

The following abbreviations are used in this manuscript:

PCLs	Pancreatic Cystic Lesions
EUS	Endoscopic Ultrasound
AI	Artificial Intelligence
CT	Computed Tomography
MRI	Magnetic Resonance Imaging
IPMNs	Intraductal Papillary Mucinous Neoplasms
MCNs	Mucinous Cystic Neoplasms
SCNs	Serous Cystic Neoplasms
SPNs	Solid Pseudopapillary Neoplasms
cNETs	cystic Neuroendocrine Tumors
CE-EUS	Contrast-Enhanced EUS
EUS-FNA	EUS-guided Fine-needle Aspiration
EUS-FNB	EUS-guided Fine-needle Biopsy
EUS-TTNB	EUS through-the-needle micro-forceps Biopsy
CEA	Carcinoembryonic Antigen
MUC	Mucin-related glycoprotein
VEGF	Vascular Endothelial Growth Factor
NGS	Next-generation Sequencing
HGD	High-grade Dysplasia
IC	Invasive Cancer
cfDNA	cell-free DNA
miRNA	microRNA
CNNs	Convolutional Neural Networks
AUC	Area Under the curve
XAI	Explainable AI
ML	Machine Learning
SVM	Support Vector Machine
